# OCTA and Doppler Ultrasound in Primary Open-Angle Glaucoma and Normal-Tension Glaucoma

**DOI:** 10.3390/life13030610

**Published:** 2023-02-22

**Authors:** Jan Lestak, Martin Fus, Marain Rybář, Antonin Benda

**Affiliations:** Faculty of Biomedical Engineering, CTU in Prague, Nám. Sítná 3105, 272 01 Kladno, Czech Republic

**Keywords:** vessel density, color Doppler imaging (CDI), visual field, primary open-angle glaucoma, normal-tension glaucoma

## Abstract

The aim of this study was to determine whether the values of vessel density (VD) and perfusion parameters in the ophthalmic artery (OA) and central retinal artery (CRA) of the same eye differ in patients with hypertensive glaucoma (HTG) from patients with normotensive glaucoma (NTG). The first group consisted of 20 HTG patients (40 eyes). Patients with HTG were verified to have primary open-angle glaucoma (POAG). The second group consisted of 20 HTG patients (40 eyes). VD was used to determine the Avanti RTVue XR from Optovue (USA). Peak systolic velocity (PSV), end-diastolic velocity (EDV) and resistance index (RI) were measured in both the central retinal artery (CRA) and ophthalmic artery (OA) using Doppler sonography on the Affinity 70G from Philips (USA). The visual field (VF) was examined by a fast threshold glaucoma program using a Medmont M700 (Australia). We showed no differences in VF, VD, PSV-OA and EDV-CRA between the two groups. Statistically significant differences between the two groups were observed in PSV in CRA (*p* = 0.04), EDV in OA (*p* = 0.009) and in RI in both CRA and OA. Other values were without significant differences in both groups. In both HTG and NTG, we observed in PSV-CRA, EDV-OA, RI-CRA and RI-OA.

## 1. Introduction

In hypertensive glaucoma (HTG), the ganglion cells of the retina and subsequently the entire visual pathway, including the visual cortex, are damaged. All ganglion cells of the retina are damaged but predominantly the magnocellular cells. High intraocular pressure (IOP) plays a major role in the damage [1,2,3,4]. In normotensive glaucoma (NTG), retinal ganglion cell nerve fiber damage occurs at the level of the anterior part of the optic nerve and chiasm [5]. Our electrophysiological examinations showed primary alteration of retinal ganglion cells in HTG. In contrast, in NTG we observed near normal retinal ganglion cell responses but significant alterations in the visual pathway [5].

These findings led us to investigate the visual cortex using functional magnetic resonance imaging (fMRI). According to electrophysiological examinations, the activity of the visual cortex should be different in both groups. We first compared the sum of sensitivities in the homolateral visual fields (fast threshold program using the Medmont instrument) in the range of 0–22 degrees in HTG patients with different degrees of damage with the results of contralateral hemisphere activity by fMRI after stimulation with a black-and-white checkerboard. Statistical results showed a moderately strong correlation between visual field changes and brain activity [5]. Similar measurements and statistical processing in NTG showed no correlation between visual field changes and activity in the visual cortex. We concluded that these would be two differing diseases [5].

Since HTG primarily involves damage to the ganglion cells of the retina, it is obvious that these patients must also have a color-vision disorder. Therefore, in the following work, we tried to determine what the fMRI activity would look like on color stimulation. For visual stimulation we used first a black-and-white and then a yellow-and-blue chessboard. We found that HTGs after yellow-and-blue stimulation showed a decrease in activated voxels to 59% of the values of black-and-white stimulation [5]. In NTG patients, the average difference in the number of activated voxels between black-and-white and blue-and-yellow stimulation was 6%. In healthy subjects, this difference was equal to 2%. This finding also reassured us of the difference between the two diseases [5].

Ischemia is likely to be the cause of functional and structural changes in NTG. This is supported by the increased frequency of optic nerve target hemorrhage, migraines, Raynaud’s phenomenon and primary vascular dysregulation syndrome (Flammer syndrome) in NTG patients compared with HTG patients [6,7,8,9,10]. It is clear from these opening sentences that these are two different diseases, although most ophthalmologists classify them as chronic simple open-angle glaucoma and treat them accordingly as hypertensive glaucoma. Therefore, we have decided to include this paper to distinguish the two diseases from each other in terms of blood supply.

It is known from the available literature that both HTG and NTG are associated with impaired blood flow in the ophthalmic artery (OA) and central retinal artery (CRA) [11,12,13]. In our previous study, we found that, in HTG, vessel density (VD) has a large effect on changes in the visual field (VF). The vascular component of density of all vessels peripapillary (PP-VDa), density of small vessels peripapillary (PP-VDs), density of all vessels in the whole image (WI-VDa) and density of small vessels in the whole image (WI-VDs) were strongly correlated with VF. There was a very weak to weak negative correlation between VF and peak systolic velocity (PSV) in the central retinal artery (CRA) and ophthalmic artery (OA). Similarly, there was a very weak correlation between VF and end-diastolic velocity (EDV) in CRA and OA. We found a weak negative correlation between VF and the resistance index (RI) in CRA and OA [14].

The situation was somewhat different in patients with NTG. PP-VDa, PP-VDs and WI-VDa correlated moderately strongly with changes in the visual field. We observed a weak correlation with VF in WI-VDs. Perfusion parameters of PSV in CRA correlated weakly positively with VF. In contrast, PSV in OA correlated weakly negatively with VF. Similarly, EDV in CRA correlated weakly positively with VF and EDV in OA correlated weakly negatively with VF. In RI, we observed a very weak correlation with changes in the visual field [15].

We were now interested to see if there was a difference between the VF, VD and flow parameters in CRA and OA for the two groups of HTG and NTG.

## 2. Materials and Methods

Two groups of patients were compared, with the same number of subjects and eyes in each group. All of them were patients of the Ophthalmology Clinic in Prague. The first group consisted of 20 HTG patients (40 eyes), of which 13 were females with an average age of 68.7 years (49–80 years) and 7 were males with an average age of 58.4 years (27–81 years). All patients with HTG were verified to have primary open-angle glaucoma (POAG). The second group consisted of 20 NTG patients (40 eyes), of which 16 were women with an average age of 56.1 years (43–79 years) and 4 were men with an average age of 60 years (51–66 years). Inclusion criteria for the study: visual acuity 1.0 with an imputed correction of less than ±3 dioptres, approximately equal changes in visual fields. All patients with HTG had an intraocular pressure (IOP) of less than 18 mmHg and no other ocular or neurological disease. The diagnosis of incipient NTG was confirmed by electrophysiological examination, and IOP was in the range of 12–16 mmHg. VD was measured using the Avanti RTVue XR from Optovue. The peak systolic velocity (PSV), end-diastolic velocity (EDV) and resistance index (RI) were measured in both the central retinal artery (CRA) and ophthalmic artery (OA) using Doppler sonography on the Affinity 70G from Philips (Andover, MA, USA). Examination was performed by a single experienced radiologist. The visual field (VF) was examined by a fast threshold program using Medmont M700 (Melbourne, Australia). The sum of sensitivities in apostilbs (asb) was evaluated in the range of 0–22 degrees of the visual field. The measured results were then compared between the two groups. The statistics were calculated using the software STATISTICA 13. All quantitative data were expressed as mean ± SD. Two sample t-tests were used to distinguish between HTG and NTG patient samples. All tests were performed at the 5% level of significance. Box plots were used for visual comparison of the samples. All tests were performed at the 5% level of significance.

## 3. Results

Statistically significant differences between the two groups were observed in the maximum systolic blood flow velocity (PSV) in the CRA (*p* = 0.04), with a higher velocity in the CRA in the HTG group of eyes. Furthermore, in the end-diastolic velocity (EDV) in OA (*p* = 0.009), there was a higher velocity in OA in the group of eyes with NTG, and in the resistive index (RI) in both CRA and OA. A higher RI was measured in the eyes with HTG. Other values were without significant differences in both groups.

We did not observe differences in the central part of the visual fields, VD and PSV-OA and EDV-CRA. Data on the average values and their standard deviations are presented in the summary table (Table 1) and, similarly, the values of statistical significance.

**Figure 1:**
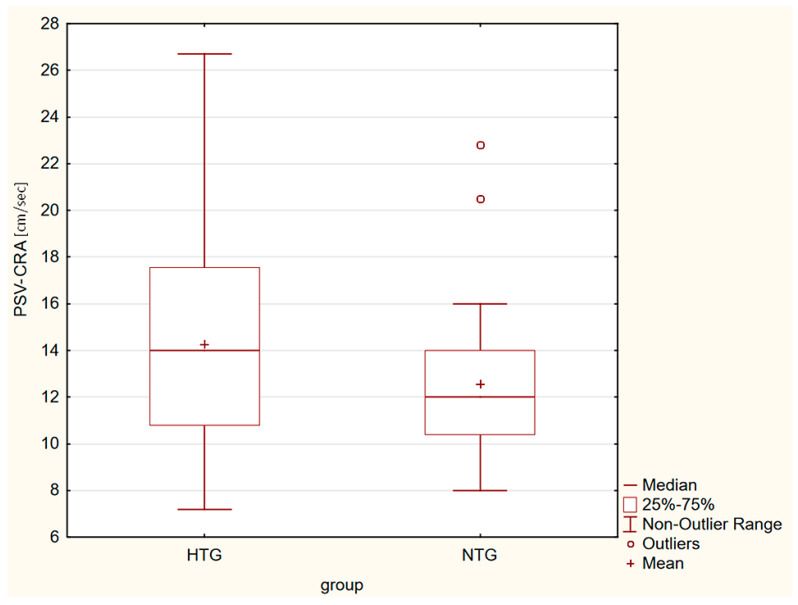
Box plot shows the differences in PSV-CRA, with the greater velocity measured in eyes with HTG.

**Figure 2:**
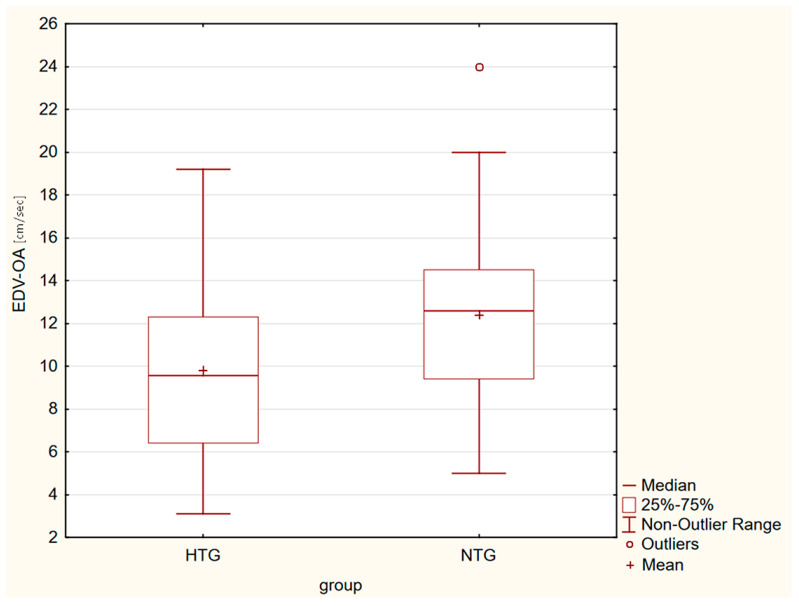
Box plot shows the differences in EDV-OA, with the greater velocity (cm/s) measured in eyes with NTG.

**Figure 3:**
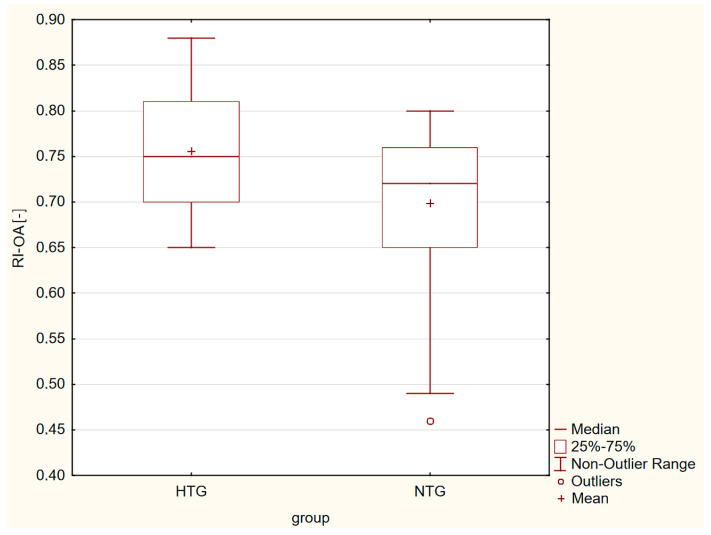
Box plot shows the differences in RI-OA, with more resistance measured in eyes with HTG.

**Figure 4:**
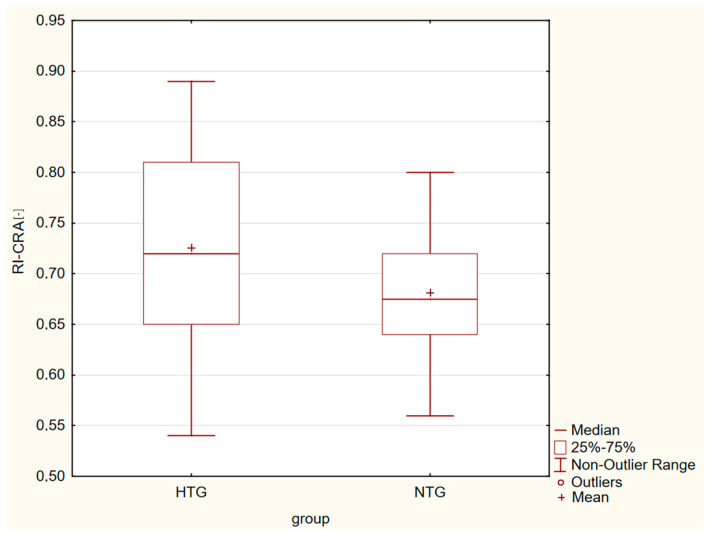
Box plot shows the differences in RI-CRA, with more resistance measured in eyes with HTG.

Box plots in Figure 1, Figure 2, Figure 3 and Figure 4 present a statistically significant difference between HTG and NTG, PSV-CRA, EDV-OA, RI-OA and RI-CRA.

## 4. Discussion

Vascular risk factors and optic nerve head perfusion are thought to influence all types of open-angle glaucoma. However, they probably have a greater influence in patients with NTG, who appear to be more susceptible to systemic vascular dysregulation and abnormal optic nerve perfusion [16,17].

Color Doppler imaging (CDI) is the most commonly used method to determine the circulatory status in ophthalmology in clinical settings. It is used to assess the blood flow velocity (PSV, EDV) in the ophthalmic vessels and also to determine their RI. A higher RI value indicates higher vascular resistance, which is indicative of a circulatory disturbance.

Vascular dysregulation leads to an unstable oxygen supply, which can result in ischemia and optic nerve damage [18,19].

OCTA is a newer non-invasive imaging modality that does not use dyes and allows qualitative and quantitative assessment of the retinal and optic nerve head vasculature.

The features observed with OCTA in eyes with glaucoma are a reduction in the density of surface vessels (VD) in the peripapillary and macular regions and complete loss of choriocapillaris in localized areas of parapapillary atrophy. These OCTA changes correlate well topographically with the functional changes seen on visual field examination and the structural changes seen on Optical Coherence Tomography (OCT) (i.e., changes in the peripapillary retinal nerve fiber layer and changes in the thickness of the inner retinal layer in the macula).

The relationship between VD, retinal nerve fiber layer and VF in HTG stages has been addressed by a number of authors. All of them found that, as glaucoma disease progresses, vessel density also becomes more impaired [20,21,22,23,24,25,26].

We were more interested in papers that compared VD in HTG and NTG.

Bojikian et al. showed that optic disc perfusion as detected by optical microangiography (OMAG) was significantly reduced in both HTG and NTG compared with the control group. No difference was observed between HTG and NTG with similar levels of VF damage [27]. Similar conclusions were reached by Lomatsch et al. [26].

Scripsema et al. found lower values for perfused capillary density (PCD) than in the control group. The decrease in PCD was statistically more significant in HTG [28].

In the HTG and NTG groups, Toshev and colleagues found significant correlations between mean peripapillary VD and global pRNFL thickness (HTG ρ = 0.71, *p* < 0.001; NTG ρ = 0.65, *p* = 0.001). This was true for all sectors except the temporal area [29].

Xu et al. found that VD in the parafoveal region was similar in both groups. However, NTG eyes had a significantly lower mean VD in the peripapillary region than HTG eyes. When different sectors of the peripapillary region were studied, the difference was still significant in most sectors (all *p* < 0.05), except for the inferotemporal sector [30].

A high correlation between VD and RNFL was also observed in the previous study. There was a moderate-to-strong correlation in eyes with IOP ≤ 20, which increased with increasing IOP to a very strong correlation in eyes with IOP > 24 mmHg [31].

We believe that capillary loss plays a very important role in both HTG and NTG, especially in the optic nerve target and its anterior region. Doppler ultrasonography confirms this assumption by detecting changes in flow parameters. We cannot yet give a clear answer as to the process by which this loss may occur.

In HTG, both IOP and the ischemic effect of glutamate may be involved in the vascular changes [32], the value of which is increased in this disease [33].

Cheng et al. sought an explanation for the higher blood viscosity in NTG, which may be related to impaired erythrocyte deformability associated with a change in erythrocyte rigidity. The higher viscoelasticity and blood viscosity in NTG patients at low velocity was due to increased erythrocyte aggregability. In addition, impaired erythrocyte deformability in NTG patients is prone to the development of distal microcirculation abnormalities. Increased blood viscosity and low efficiency of blood oxygen transport may lead to optic nerve hypoperfusion in NTG patients [34].

In order for a given amount of blood to flow through a given area, the speed of blood must increase when the bloodstream is disturbed. This was also found in our group of eyes with HTG (PSV-CRA).

Similar changes occurred in the RI values in OA and CRA. A greater resistance, which was also statistically significant, was found in HTG.

EDV in OA were similarly different. A higher final velocity in diastole was observed in NTG. Here too, the difference was statistically significant.

PSV reflects the strength of vessel perfusion, whereas EDV reflects the blood perfusion of distal organs and is a sensitive indicator of increased downstream impedance. RI is considered to reflect vascular resistance peripheral to the location where the measurement is made but is not equivalent to vascular resistance because it depends on both vascular resistance and vascular compliance; only at high vascular compliance is RI an adequate measure of vascular resistance. However, the association of lower EDV with higher RI could be explained by increased vascular resistance [35,36], and changes in resistance affect diastolic blood flow velocity more than systolic velocity [37], which further aggravates the ischemia of organs.

There is very little literature available on issues similar to those addressed in this paper. The vast majority of them do not distinguish the difference between HTG and NTG and classify both groups under primary open-angle glaucoma. Therefore, we present here only three that distinguished HTG from NTG.

Perfusion parameters between HTG and NTG were also compared by Wiermann et al. Both glaucoma groups showed statistically significant decreases in PSV and EDV in CRA and short posterior ciliary arteries compared with the control subjects. HTG when compared with NTG and normal subjects showed statistically significant decreases of EDV and statistically significant increases of RI in long posterior ciliary arteries. In addition, compared with normal subjects, HTG patients showed statistically significant increases of RI in both OA and short posterior ciliary arteries [38].

Zhong et al. compared differences in color Doppler imaging (CDI) and visual evoked potential (P-VEP) testing between HTG and NTG patients.

Patients with NTG and HTG had lower EDV and higher RI in the OA, CRA and short posterior ciliary arteries compared with control subjects, and HTG also had lower PSV in the OA and CRA compared with control subjects. There was no significant difference in blood flow velocities and RI of all retrobulbar vessels between NTG and HTG patients. P100 latency in VEP was delayed and P100 amplitude was decreased in NTG and HTG patients compared with controls. There was no significant difference in P100 latency and amplitude between NTG and HTG patients. RI-OA and short posterior ciliary arteries were negatively correlated with mean deviation (MD) of visual field values in NTG and HTG patients. RI-OA was positively correlated with pattern standard deviation (PSD) of visual field values in NTG and HTG patients. Mean defect (MD) values were negatively correlated with P100 latency time in NTG and HTG patients. RI-OA was positively correlated with P100 latency time in NTG and HTG patients. RI-OA was negatively correlated with P100 amplitude in HTG patients. There was no significant difference in CDI and P-VEP parameters between NTG and HTG patients. Some CDI parameters were correlated with P-VEP parameters in NTG and HTG patients [39].

Our results are almost identical to the Leuven Eye Study, which also measured flow parameters in HTG and NTG. They found that the PSV in the CRA was higher in HTG than in NTG. Similarly, RI-CRA and RI-OA were statistically significantly higher in HTG than in NTG [40].

We recorded statistically significant differences between the two groups in the maximum systolic blood flow velocity in the CRA (*p* = 0.04), with a higher velocity in the CRA in the HTG group of eyes, and in the end-diastolic velocity in OA (*p* = 0.009), with a higher velocity in CRA in the group of eyes with NTG, and in the resistive index in both CRA and OA. A higher RI was measured in the eyes with HTG. Other values were without significant differences in both groups.

We did not observe statistically significant changes between HTG and NTG in the sum of sensitivities in the central part of the visual fields, VD and PSV-OA, and EDV-CRA.

Higher values of the sum in apostilbs were in HTG. The NTG is characterized by changes in the central part of the visual field in contrast to the HTG [41,42,43,44], and therefore the sensitivity values in our case were also lower for NTG.

This work also confirms the distinctness of HTG from NTG, which we also confirmed by examining functional and structural parameters [5].

Our work has its limits. First, we have to mention the missing data on antiglaucoma drug treatment in both groups. This work was done by comparing two previous studies where we did not evaluate the possible effect of antiglaucomatous drugs mainly on perfusion parameters in CRA and OA [14,15]. The question is whether local antiglaucoma treatment can affect, e.g., perfusion parameters in OA. However, we have to emphasize that both groups were at approximately the same stage of the disease, namely early. The Medmont perimeter does not use indices such as mean sensitivity, mean defect and others, but overall and pattern defect. Therefore, we used the sum of the sensitivities in the central part of the visual fields to compare the two groups. The groups did not differ in the sensitivity of the visual fields. Another shortcoming may be the wide age range. For HTG, 27–81 years, and, for NTG, 43–79 years. However, each subject was compensated by the cardiovascular side. Despite these shortcomings, we think that a difference in the evaluated and statistically proven parameters between the two groups does exist.

## 5. Conclusions

Our results showed that there were no differences in VF (its central part), VD, PSV-OA and EDV-CRA in the early stages between HTG and NTG. The groups differed in PSV-CRA, EDV-OA, and RI-CRA and RI-OA. These differences may also point to two distinct diagnostic groups.

## Figures and Tables

**Table 1:** Summary data compared in both groups.

	HTG (40 Eyes)	NTG (40 Eyes)	*p*-Value
	Mean ± SD		
age	67.15 ± 12.86	66.68 ± 9.17	*p* = 0.855
VF [asb]	2022.48 ± 154.30	1990.84 ± 294.07	*p* = 0.550
PP-VDa [%]	550.08 ± 5.08	55.07 ± 5.38	*p* = 0.992
PP-VDs [%]	48.96 ± 5.15	48.72 ± 5.36	*p* = 0.844
WI-VDa [%]	52.81 ± 4.35	53.03 ± 3.97	*p* = 0.815
WI-VDs [%]	46.49 ± 4.31	45.52 ± 7.26	*p* = 0.471
PSV-OA [%]	40.11 ± 12.83	41.22 ± 9.20	*p* = 0.662
PSV-CRA [cm/s]	14.27 ± 4.17	12.55 ± 3.09	*p* = 0.042
EDV-OA [cm/s]	9.82 ± 4.30	12.39 ± 4.22	*p* = 0.009
EDV-CRA [cm/s]	4.17 ± 2.31	3.98 ± 1.09	*p* = 0.638
RI-OA [-]	0.76 ± 0.07	0.70 ± 0.08	*p* = 0.001
RI-CRA [-]	0.73 ± 0.09	0.68 ± 0.06	*p* = 0.001

VF—visual field (asb = apostilbs), PPVDa—density of all vessels peripapillary, PPVDs—density of small vessels peripapillary, WIVDa—density of all vessels in the whole image, WIVDs—density of small vessels in the whole image, PSV—peak systolic velocity, EDV—end-diastolic velocity, RI—resistance index, CRA—central retinal artery, OA—ophthalmic artery.

## Data Availability

Not applicable.
